# New Hydroxycinnamic Acid Esters as Novel 5-Lipoxygenase Inhibitors That Affect Leukotriene Biosynthesis

**DOI:** 10.1155/2017/6904634

**Published:** 2017-06-07

**Authors:** Luc H. Boudreau, Grégoire Lassalle-Claux, Marc Cormier, Sébastien Blanchard, Marco S. Doucet, Marc E. Surette, Mohamed Touaibia

**Affiliations:** Department of Chemistry and Biochemistry, Université de Moncton, Moncton, NB, Canada E1A 3E9

## Abstract

Leukotrienes are inflammatory mediators that actively participate in the inflammatory response and host defense against pathogens. However, leukotrienes also participate in chronic inflammatory diseases. 5-lipoxygenase is a key enzyme in the biosynthesis of leukotrienes and is thus a validated therapeutic target. As of today, zileuton remains the only clinically approved 5-lipoxygenase inhibitor; however, its use has been limited due to severe side effects in some patients. Hence, the search for a better 5-lipoxygenase inhibitor continues. In this study, we investigated structural analogues of caffeic acid phenethyl ester, a naturally-occurring 5-lipoxygenase inhibitor, in an attempt to enhance the inhibitory activity against 5-lipoxygenase and determine structure-activity relationships. These compounds were investigated for their ability to attenuate the biosynthesis of leukotrienes. Compounds **13** and **19**, phenpropyl and diphenylethyl esters, exhibited significantly enhanced inhibitory activity when compared to the reference molecules caffeic acid phenethyl ester and zileuton.

## 1. Introduction

The 5-lipoxygenase (5-LO) enzyme catalyzes the first two steps of the conversion of arachidonic acid into leukotrienes (LTs) [1]. LTs are potent lipid mediators that actively participate in numerous inflammatory diseases such as atherosclerosis [2], asthma [3], arthritis [4], and several types of cancers [5–7]. As the cornerstone of the LT biosynthesis pathway, it is not surprising that the regulation of 5-LO activity has been the focal point of many therapeutic approaches in recent years (reviewed in [8–10]).

**Figure 1:**
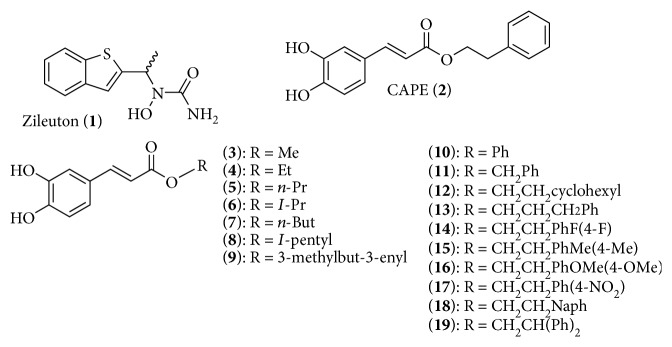
Zileuton (**1**), CAPE (**2**), and CAPE analogues investigated in the present study.

Currently, zileuton (**1**, Figure 1) (Zyflo®) remains the only clinically used and approved 5-LO inhibitor. It is primarily prescribed to help alleviate chronic asthma symptoms [11, 12]. However, possible liver toxicity has been associated with long-term intake of the drug [13]; thus, the search for better 5-LO inhibitors with less side effects continues. Amongst the new wave of 5-LO inhibitors are naturally occurring molecules derived from plant extracts [14]. Honeybee propolis is a resinous substance composed of 50% plant resins, 30% wax, 10% essential and aromatic oils, 5% pollens, and 5% of other organic substances (reviewed in [15]). Propolis has been shown to exhibit various beneficial biological properties, such as anti-inflammatory, antibacterial, antiviral, and anticancer effects [16–18]. Caffeic acid phenethyl ester (CAPE (**2**, Figure 1)) is one of the major bioactive components of honeybee propolis [19]. We and others have shown that CAPE (**2**) and some structural analogues are potent inhibitors of the 5-LO pathway [20–23].

In this study, CAPE (**2**) and some structural analogues of the ester moiety were synthesized in an attempt to enhance inhibitory activity against 5-LO and determine structure-activity relationships. As shown in Figure 1, two families of CAPE analogues were investigated. One is a series of aliphatic esters and the second is substituted aryl esters. These compounds were investigated for their ability to attenuate the biosynthesis of LTs, their specificity to the 5-LO enzyme, and their potency as inhibitors in a complex environment such as freshly isolated human blood. In summary, the aryl ester compounds **13** and **19** exhibited the most potent inhibitory activity showing significantly better inhibition when compared to the reference molecules CAPE (**2**) and zileuton (**1**).

## 2. Methods

### 2.1. CAPE Analog Synthesis

As recently reported [24], alkyl esters (**3**–**9**) were synthesized in one step by Fisher esterification with selected alcohols and caffeic acid. Aryl esters (**10**–**19**) were synthesized by esterification of (*E*)-caffeoyl chloride diacetate with selected alcohols followed by a de-*O*-acetylation.

### 2.2. HEK293 Cell Stimulation and Measurement of 5-LO Products

HEK293 cells expressing both 5-LO and the 5-lipoxygenase activating protein (FLAP) were generated as previously reported [25–27]. For cell stimulation of 5-LO products, transfected HEK293 cells were collected following trypsinization and washed and the cell pellet was resuspended in Hank's balanced salt solution (HBSS, Lonza) containing 1.6 mM CaCl_2_ at a concentration of 5 × 10^5^ cells/ml. Cells were preincubated with each compound at the indicated concentration for 5 min at 37°C. Cells were then stimulated for 15 minutes at 37°C with the addition of 10 *μ*M calcium ionophore A23187 (Sigma-Aldrich) and 10 *μ*M arachidonic acid (Cayman Chemical). Stimulations were stopped by adding 0.5 volume of cold MeOH:MeCN (1 : 1) containing 100 ng/ml of 19-OH prostaglandin B_2_ (PGB_2_) as internal standard. Samples were then processed and 5-LO products were analyzed by reversed-phase high-performance liquid chromatography (RP-HPLC) as described previously [20, 28].

### 2.3. Polymorphonuclear Leukocyte and Platelet Isolation

Polymorphonuclear leukocytes (PMNL) were isolated as previously described [20, 29]. Briefly, whole blood was collected (with anticoagulant citrate dextrose (ACD)) and then centrifuged at 500 *g* for 10 min. Platelet-rich plasma (PRP) was set aside for platelet isolation (see below), and erythrocytes were removed by dextran sedimentation. After a centrifugation step at 900 *g* × 20 min at room temperature on a lymphocyte separation medium cushion (density, 1.077 g/ml) (Wisent), PMNL were obtained (>96%) followed by hypotonic lysis of the remaining erythrocytes. PMNL were counted and resuspended at 10^7^ cells/ml in HBSS supplemented with 1.6 mM CaCl_2_. PRP was processed as previously described for platelet isolation [30]. Briefly, PRP was centrifuged at 400 *g* for 2 min at room temperature to remove the remaining erythrocytes. The supernatant was then centrifuged at 1300*g* for 10 min to pellet platelets. Cells were resuspended in Tyrode Buffer pH 7.4 (134 mM NaCl, 2.9 mM KCl, 0.34 mM Na_2_HPO_4_, 12 mM NaHCO_3_, 20 mM HEPES, 1 mM MgCl_2_, 5 mM glucose, and 0.5 mg/ml BSA) at 3 × 10^8^ cells/ml in the presence of 5 mM CaCl_2_.

### 2.4. Biosynthesis of 5-Lipoxygenase Products by PMNL

Suspended PMNL (10^7^ cells/ml) were incubated at 37°C with adenosine deaminase (0.3 U/ml, Sigma-Aldrich) and test compounds at indicated concentrations 5 min before stimulation. To initiate stimulation, 1 *μ*M of thapsigargin (Sigma-Aldrich) was added to cells which were then incubated for 15 min at 37°C [31]. Two volumes of MeOH:MeCN (1 : 1) containing the internal standard PGB_2_ (100 ng/ml) was then added, and samples were processed for RP-HPLC analysis as described above.

### 2.5. Arachidonic Acid Release

Quantification of arachidonic acid release from cellular membranes was performed as previously described [31, 32]. Briefly, freshly isolated PMNL (10^7^ cells/ml, in HBSS containing 1.6 mM CaCl_2_ and 0.1% BSA) were incubated with adenosine deaminase (0.3 U/ml) and test compounds for 5 min at 37°C. Stimulation was initiated with the addition of 1 *μ*M thapsigargin, followed by incubation at 37°C for 5 min. The reactions were stopped with the addition of two volumes of cold MeOH and 300 ng of arachidonic acid-d_8_ (Cayman Chemicals) as internal standard. The samples were stored at −20°C overnight and then centrifuged the next day at 1000 *g* for 10 min. The supernatants were diluted with four volumes of acidified water (0.1%) then processed on an octadecyl (C18) column. Samples were eluted with the addition of 3 ml of MeOH and dried under nitrogen. Pentafluorobenzylesters were prepared with the addition 50 *μ*l N,N-diisopropylethylamine (20% in acetonitrile) and 50 *μ*l of 2,3,4,5,6-pentafluorobenzylesters (20% in acetonitrile). Samples were heated for 40 min at 40°C, then dried under nitrogen and resuspended in 100 *μ*l of hexane. Samples were quantified by negative ion chemical ionization gas chromatography/mass spectrometry using TraceGC ultra column (Thermo) and a Polaris Q mass spectrometer (Thermo).

### 2.6. Platelet Activation

Platelets (3 × 10^8^ cells/ml) were preincubated with various test compounds at indicated concentrations for 5 min at 37°C. Platelet activation was initiated with the addition of 10 *μ*M of calcium ionophore A23187 (Sigma-Aldrich) followed by a 5 min incubation time at 37°C. Reactions were stopped with the addition of two volumes of cold MeOH:MeCN (1 : 1) containing PGB_2_ (100 ng/ml) as internal standard. Samples were stored overnight at −20°C and were then centrifuged, the supernatant was dried under nitrogen, and the samples were resuspended in 30% methanol and analyzed by RP-HPLC as indicated above.

### 2.7. Ex Vivo Whole Blood Stimulation

Ex vivo whole blood stimulation for the biosynthesis of 5-LO products was performed as previously reported [20]. Briefly, blood was collected in tubes containing heparin as anticoagulant. Each compound tested or its diluent control (DMSO, 0.5%) was added to 1 ml of heparinized blood and incubated for 5 min in a water bath at 37°C. Stimulation was initiated with the addition of 125 *μ*l of 40 mg/ml of opsonized zymosan, samples were gently vortexed and incubated at 37°C for 30 min. Samples were then centrifuged at 960 *g* for 10 min at 4°C. Plasma (350 *μ*l) was removed and added to tubes containing 1.2 ml of CH_3_OH:CH_3_CN (1 : 1) containing 100 ng/ml of PGB_2_ as internal standard. Samples were stored overnight at −20°C, then centrifuged at 500 *g* for 10 min. The supernatants were dried under nitrogen, resuspended in 30% MeOH, and analyzed by RP-HPLC as described above.

### 2.8. Molecular Docking

Molecular docking was undertaken with the help of AutoDock 4.0, Autogrid [33], and AutoDock Tools [34]. The standard AutoDock protocol was followed unless otherwise noted. Ligands were drawn and then processed with AutoDock Tools for charge and rotatable bonds assignment. 5-LO crystal structure chosen for docking is PDB ID: 3O8Y [34], which is a “stable-5-LOX.” To enable crystallization, several mutations are present in the noncatalytic domain and a small 3 residue sequence in the catalytic domain is replaced from KKK to ENL. The mutations maybe affect the structure, but “stable-5-LOX” catalytic activity was not affected [34]. The protein was prepared with AutoDock Tools. Water molecules were removed, polar hydrogens added, and charges assigned. The grid box used a default spacing of 0.375 with a bounding box of 60, 66, and 60 and a grid center of −2.24, 25.69, and −0.94, in both cases X, Y, and Z coordinates. As for the docking settings, 100 runs were completed per ligand and defaults were kept with exceptions for the following values: ga_pop_size 5000, ga_num_evals 100,000,000, ga_num_generations 500,000, and sw_max_its 5000. For analysis, AutoDock Tools (Schrödinger Release; Maestro, version 10.6) and LigPlot^+^ [35] were used. Results were clustered with a maximum of 2.00 Å RMSD, and the largest cluster with the lowest binding energy was chosen.

### 2.9. Statistical Analyses

Statistical analysis and graph design were performed with GraphPad Prism 5 software (GraphPad Software, San Diego, California). IC_50_ values were calculated from a sigmoidal concentration-response curve-fitting model and are expressed as means with 95% confidence intervals. All other data are expressed as mean ± SEM. One-way ANOVA with Dunnett's multiple comparison test (*p* < 0.05) were performed to determine significant difference from controls.

## 3. Results

### 3.1. Compound Synthesis

CAPE (**2**) is a simple molecule for which several biological activities have been reported. In continuation of previous work on the development of new 5-LO inhibitors based on CAPE (**2**), the synthesis of CAPE analogues and the testing of 5-LO inhibition were undertaken. With these series of compounds, the importance of the nature of the ester moiety was explored (Figure 1). Indeed, this moiety is crucial for the inhibition of 5-LO by CAPE (**2**) as demonstrated previously [20].

The first series with the alkyl esters allowed the evaluation of the presence and the nature of an alkyl chain (**3–9**). The alkyl chains vary from the simple methyl ester (**3**), proceeding to the 3-methyl-3-enyl ester (**9**) representing a mimic of CAPE (**2**) with methyl group and a single double bond. The second series allowed us to explore the effect of the presence of an aryl and the effect of a monosubstitution on this aryl (**10–19**). The effect of the presence and the length of a linker between the oxygen and the aryl group was also investigated (Figure 1). The two series of compounds were synthesized by two different strategies. The alkyl series was obtained in a single step following a single esterification of caffeic acid and an alcohol to give the desired ester. The aryl esters were synthesized in three steps with the (*E*)-caffeoyl chloride diacetate, obtained by the Vilsmeier-Haack adduct derived from thionyl chloride and N,N-dimethylformamide as catalyst, and the appropriate alcohol. The structures of all synthesized esters are summarized in Figure 1.

### 3.2. Biosynthesis of 5-LO Products

**Figure 2:**
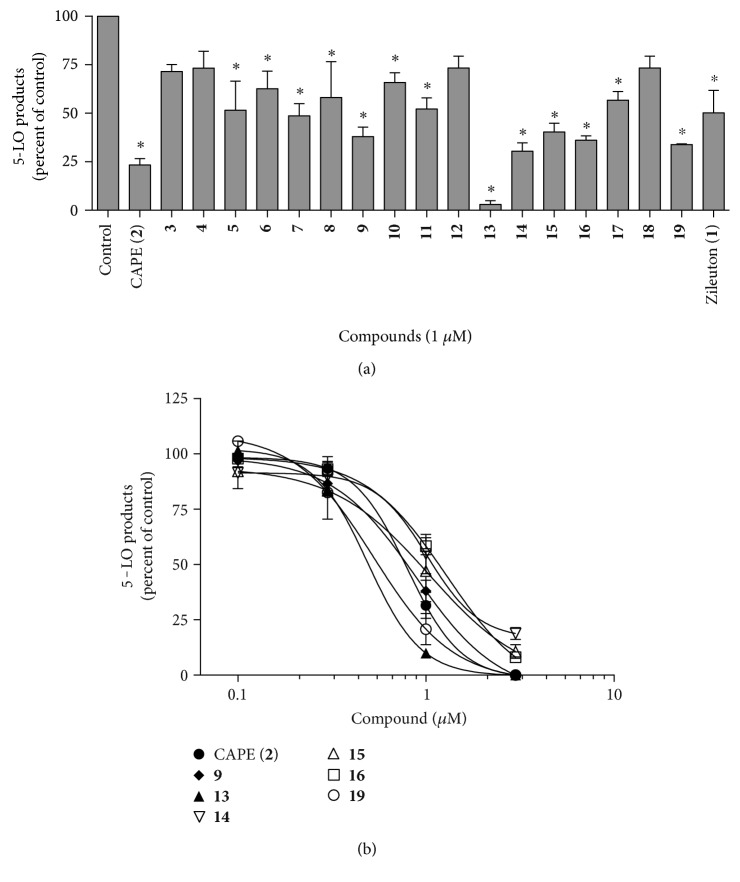
Effect of the test compounds on the biosynthesis of 5-LO products in transfected HEK293 cells (a) and thapsigargin-stimulated PMNL (b). (a) HEK293 cells expressing both 5-LO and FLAP were incubated in presence of the indicated compounds (1 *μ*M) or their diluent (control, 0.5% DMSO) for 5 min, then biosynthesis of 5-LO products was initiated with the addition of 10 *μ*M calcium ionophore A23187 and 10 *μ*M arachidonic acid for 15 min. After stimulation, reactions were stopped and samples were processed for analysis of 5-LO products by RP-HPLC. Total 5-LO products measured represent the sum of LTB_4_, its trans isomers, and 5-hydroxyeicosatetraenoic acid. (b) Freshly isolated human PMNL were incubated in presence of test compounds (or their diluent control, DMSO 0.5%) at the indicated concentrations for 5 min. Biosynthesis of the 5-LO products was initiated with the addition of 1 *μ*M of thapsigargin, followed by a 15 min incubation period at 37°C. The stimulation was then stopped and samples were processed for RP-HPLC analysis and total 5-LO products quantification. Total 5-LO products measured represent the sum of LTB_4_, its trans isomers, 20-COOH- and 20-OH-LTB_4_ and 5-hydroxyeicosatetraenoic acid. Data are expressed as means ± SEM of at least 3 independent experiments. ^∗^Different from control, as determined by one-way ANOVA with Dunnett's multiple comparison test (*p* < 0.05).

To evaluate CAPE analogues as potential inhibitors of the biosynthesis of 5-LO products, a first series of experiments was performed in HEK293 cells expressing both 5-LO and FLAP as previously described [20, 26, 27, 36]. HEK293 cells were first preincubated with the test compounds (1 *μ*M), then stimulated in the presence of arachidonic acid and calcium ionophore A23187 to induce the biosynthesis of 5-LO products (Figure 2(a)). Compounds that showed similar or better inhibitory activity than the reference compound CAPE (**2**), or decreased the biosynthesis of 5-LO products by more than 50% (when compared to the control), were selected for subsequent testing. Based on these criteria, compounds **9**, **13, 14**, **15**, **16**, and **19** were selected for subsequent analyses. Of importance, zileuton (**1**), the only 5-LO inhibitor currently used clinically, was less effective than the selected compounds (Figure 2(a)). It should be noted that many of the compounds that were not selected for further study are nevertheless inhibitors of 5-LO that may well have IC_50_ values inferior to 10 *μ*M. If the selected compounds do not perform well in future pharmacokinetic studies, these other compounds may nevertheless be interesting preclinical candidates that could be reinvestigated.

Having selected the most potent compounds, a second series of experiments measuring the dose response of the inhibition of 5-LO product biosynthesis was conducted using freshly isolated human PMNL to determine the IC_50_ values for each test compound (Figure 2(b) and Table 1). Compounds **13** (IC_50_ = 0.49 *μ*M) and **19** (0.53 *μ*M) had a significantly lower IC_50_ value than CAPE (**2**) (0.79 *μ*M, Table 1). Compounds **9** (0.87 *μ*M), **14** (1.01 *μ*M), **15** (1.04 *μ*M), and **16** (1.29 *μ*M) had IC_50_ values that were similar to CAPE (**2**) (Table 1). Of importance, compound 13 (the most potent inhibitor of 5-LO product biosynthesis) did not induce early apoptosis of PMNL as indicated by annexin V labelling (see Supplementary Figure S1 available online at https://doi.org/10.1155/2017/6904634).

### 3.3. Molecular Docking

A total of 19 esters were docked to 5-LO in this study. From the obtained docking results, the binding energy was determined for each ligand as well as hydrogen bonds and *π*-*π* interactions (Table 2). The most stable ligand, with the lowest binding energy, was (**17**) with a binding energy of −10.91 kcal/mol and (**18**), −10.61 kcal / mol. The least stable ligand was (**4**) with a binding energy of −6.15 kcal/mol.

The pose of each ligand could be classified into 3 larger groups with a handful of subdivisions. Inside these groups, the catechol moiety often has only a slight variation between each molecule. For the monosubstituted ester moiety, the variation in position is often larger, depending on the characteristics of the substitution.

**Figure 3:**
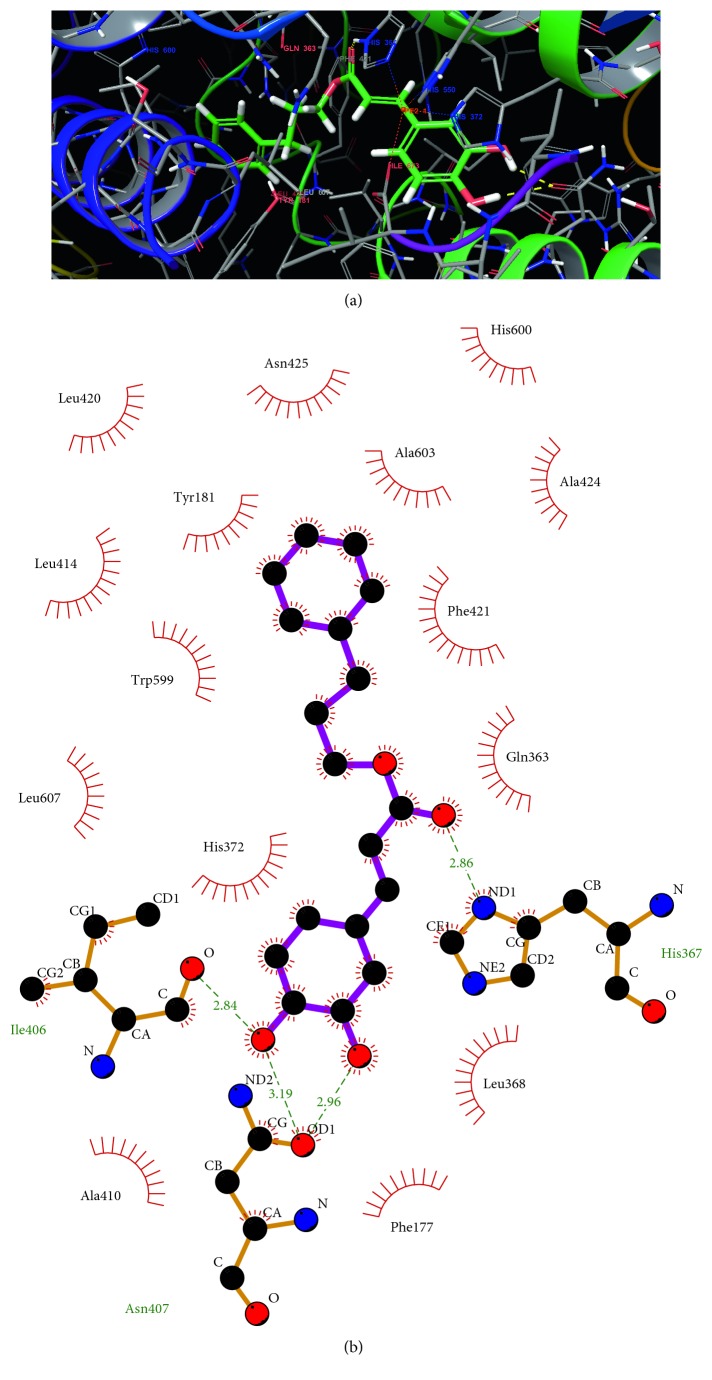
(**13**) docking (a); (**13**) with AutoDock displayed in Maestro and LigPlot (b).

The first group has all molecules generating double hydrogen bonds with residue Asn407 and a single hydrogen bond with His367. This group is formed by (**12**), (**13**), (**15**), (**16**), (**17**), (**18**), and CAPE (**2**). To interact with these two residues, the ligand must complete a “cage” around the iron atom with the coordinating residues. This cage would block access to the iron atom of any other molecule. A minimum of 3 hydrogen bonds are formed, with the possibility of more being formed depending on the monosubstituted ester moiety. This type of pose would make it hard to displace the ligand once in place. An advantage of this pose is that His367 is an iron-coordinating residue [1, 37], and it has been suggested that His367 may serve as a replaceable iron ligand [1]. While most atoms in the molecules appear just beyond the reach of interaction with the iron, this would make this group perfectly placed to coordinate or interact with the iron atom after a small shift of the caffeic acid moiety and possibly replace His367.  Figure 3 shows (**13**) in the active site as well as all interactions. These 7 ligands are the most stable of the series with the exception of (**19**) occupying the fourth place. The average binding energy for this first group is −9.23 kcal/mol, compared to −7.30 kcal/mol for the remaining molecules.

A subgroup of the first group, (**14**) and (**19**), occupy a similar pose to the first group, but they do not form the same hydrogen bonds. In the case of (**19**), the molecule is “inverted” when compared to all other molecules in this series. Its hydroxyl groups point towards the end of the cavity versus “pointing” toward the iron atom. This might be due to the large size of its double phenyls. One of its double phenyls overlaps with the caffeic acid moiety of the first group of molecules, while the second phenyl positions itself near Thr364. For both molecules in this subgroup, they still appear to form the “cage.”

A second subgroup of the first group contains only zileuton (**1**). It has a smaller size and a different shape than the other ligands making certain aspects less evident to compare. When compared with the other groups, zileuton's (**1**) pose overlaps the most with the molecules in the first group, where its aromatic ring lines up relatively well and its linear chain generally follows the linker.

The second group is only made of (**3**), which is positioned far away from the iron, at the end of the pocket near Leu420 with the linear part of the molecule at the end of the pocket. This leaves no possibility for (**3**) to interact directly with the iron atom without travelling inside the pocket.

The last group is made up of 8 molecules (**4**–**11**), with 4 minor subgroups. The only difference between theses minor subgroups is a slightly different placement and rotation of the catechol moiety of the molecule. The ligands are as follows, with each minor subgroup separated by a semicolon: (**4**), (**5**), (**6**) and (**7**); (**8**) and (**9**); (**10)**; (**11)**. These compounds are distant from the iron atom and would need some movement for the possibility to interact with the iron. Compared to the other molecules, they are the least stable with an average binding energy of −7.17 kcal/mol versus the −8.71 kcal/mol of the remaining molecules.

Overall, the most prevalent hydrogen bond is with Tyr181, present in 8 out of 17 molecules. Hydrogen bonds with Gln363, His367, and Asn425 are present with 7 molecules, and 6 molecules make hydrogen bonds with Asn407. Tyr181, Gln363, and Asn425 form bonds with the molecules that are less stable. Asn407, His367, and to a certain extent Phe421 seem like the most import residues since the majority of the better scoring ligands, mostly comprised of molecules from the first group, form hydrogen bonds with Asn407 and His367. For a portion of the best-ranked ligands, a *π*-*π* interaction is observed with Phe421.

From the images generated by Ligplot+, almost all atoms are under hydrophobic contact. Under visual inspection, most of the best scoring ligands occupy a good portion of the active site cavity and they all seem to block any access to the iron atom. In the case of (**19**), the entire cavity is almost filled. The lesser scored ligands, mostly the last group, appear to occupy the end of the cavity, near Leu420, and mostly leave a large opening around the iron atom. This is especially true for (**3**).

### 3.4. Arachidonic Acid Liberation from Cells

**Figure 4:**
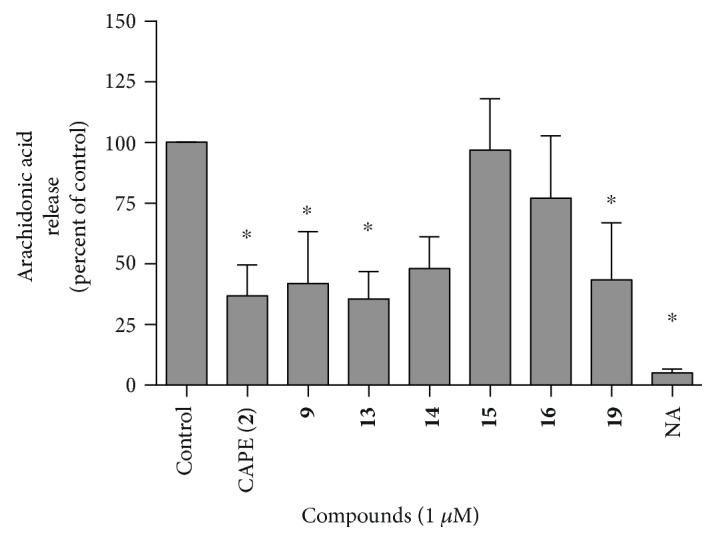
The impact of test compounds on the liberation of arachidonic acid in stimulated PMNL. Human PMNL were incubated with test compounds (1 *μ*M) or their diluent (control, DMSO 0.5%) for 5 min. Stimulation was then initiated with the addition of 1 *μ*M thapsigargin, followed by an incubation at 37°C for 5 min. The reactions were stopped with the addition of two volumes of cold MeOH and 300 ng of arachidonic acid-d_8_, then samples were stored overnight at −20°C. Free arachidonic acid was first extracted on octadecyl columns, then pentafluorobenzylesters of arachidonic acid were prepared and samples were measured on GC-MS. Spontaneous release of AA was also measured in absence of thapsigargin (nonactivated, NA). ^∗^Different from control as determined by one-way ANOVA with Dunnett's multiple comparison test (*p* < 0.05). Data are expressed as means ± SEM of at least 3 independent experiments.

A limiting factor in the generation of 5-LO products in PMNL is the bioavailability of its substrate, arachidonic acid, which is acylated in cell membrane phospholipids and must be released by a phospholipase A_2_. Since both CAPE (**2**) and zileuton (**1**) were previously shown to inhibit the liberation of cellular arachidonic acid [31, 38], the test compounds were evaluated to determine if they would also inhibit arachidonic acid release, thus partially contributing to the decrease of 5-LO products generated by the cells. As shown in Figure 4, CAPE (**2**), **9**, **13**, and **19** significantly inhibited cellular arachidonic acid release when compared to the control (0.5% DMSO, vehicle). On the other hand, compounds **14**, **15**, and **16** failed to significantly inhibit the release of cellular membrane arachidonic acid, although compound 14 approached significance (Figure 4).

### 3.5. Ex Vivo Biosynthesis of 5-LO Products

**Figure 5:**
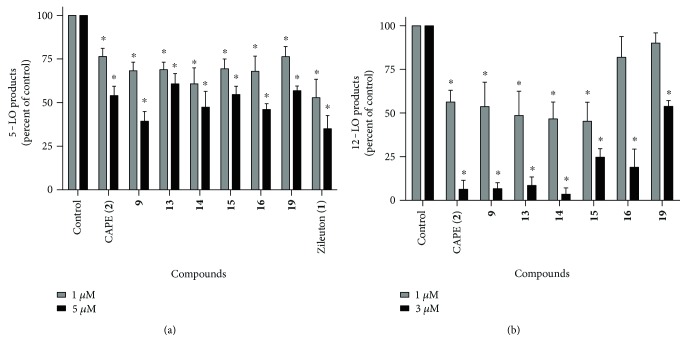
The inhibitory effects of the test compounds of the biosynthesis of 5-LO products in stimulated whole blood (a) and the biosynthesis of 12-lipoxygenase products in platelets (b). (a) Whole blood was incubated in presence of various compounds (1 *μ*M and 5 *μ*M) or with their diluent (control, DMSO 0.5%) for 5 min, then stimulated with opsonized zymosan (5 mg/ml) for 30 min at 37°C. Blood was then centrifuged and plasma was collected and added to 3.5 volumes of MeOH:CH_3_CN (1 : 1). Samples were processed for analysis by RP-HPLC for total 5-LO products quantification. Total 5-LO products measured represent the sum of LTB_4_, its trans isomers, 20-COOH and 20-OH-LTB_4_, and 5-hydroxyeicosatetraenoic acid. (b) Platelets were incubated for 5 min in presence of test compounds (1 *μ*M and 3 *μ*M) then biosynthesis of 12-HETE was initiated with the addition of 10 *μ*M of calcium ionophore A23187. Reactions were stopped with the addition of two volumes of MeOH:MeCN (1 : 1 *v*/*v*). Samples were processed for analysis by RP-HPLC for 12-hydroxyeicosatetraenoic acid (12-HETE) quantification. ^∗^Different from control as determined by one-way ANOVA with Dunnett's multiple comparison test (*p* < 0.05). Data are expressed as means ± SEM of at least 3 independent experiments.

Since selected compounds inhibit the biosynthesis of 5-LO products in both fresh human PMNL and the HEK293 cell line expressing the machinery for leukotriene generation, the ability of the compounds to inhibit 5-LO product biosynthesis in a more physiological and complex environment was measured. Freshly obtained human blood was preincubated in presence of test compounds (1 *μ*M) and then 5-LO product biosynthesis was initiated with addition of zymosan. As shown in Figure 5(a), all compounds that had been selected after screening in HEK293 cells significantly decreased 5-LO product biosynthesis in whole blood at 1 *μ*M and 5 *μ*M. Of importance, in addition to thapsigargin stimulation of isolated PMNL, the use of zymosan in this series of experiments showed that these compounds inhibit the biosynthesis of 5-LO products induced by different stimuli, and in a complex matrix.

### 3.6. Inhibition of Platelet-Type 12-Lipoxygenase

Having identified compounds that inhibit the biosynthesis of lipid mediators generated via the 5-LO pathway, their ability to inhibit platelet 12-lipoxygenase (12-LO) was assessed to determine whether they show specificity. Platelets do not express 5-LO but express high levels of the 12-LO enzyme which is responsible for the conversion of arachidonic acid into 12-hydroxyeicosatetraenoic acid (12-HETE), an inflammatory lipid mediator implicated in vascular permeability. Compounds CAPE (**2**), **9**, **13**, **14**, and **15** significantly inhibited the biosynthesis of 12-HETE (Figure 5(b)) at a concentration of 1 *μ*M with near-complete inhibition at 3 *μ*M. Although they showed good inhibition of 5-LO, compounds **16** and **19** failed to significantly affect the biosynthesis of 12-HETE at a concentration of 1 *μ*M although significant inhibition was measured at a concentration of 3 *μ*M.

## 4. Discussion

Inflammatory lipid mediators, such as LTs generated via the 5-LO pathway, are necessary for the body's defense against pathogens. However, the production of LTs has also been associated with several inflammatory diseases such as asthma [3], arthritis [4], atherosclerosis [2], and some types of cancers [5, 6]. Therefore, physiological and pharmacological regulation of LT biosynthesis remains a focal point of numerous research efforts investigating the control of inflammatory diseases [8, 9, 39]. To this day, zileuton (**1**) (Zyflo) remains the only 5-LO inhibitor approved for use by clinicians to help alleviate symptoms associated with asthma [11, 12]. However, serious side effects have been observed with the use of this drug in some patients [13], thus prompting new and innovative approaches in drug design and development to regulate the production of LTs.

In this study, we synthesized a new series of structural analogue of CAPE (**2**), a natural occurring compound found in honeybee propolis previously reported as an inhibitor of lipoxygenase pathways [18, 22, 31]. Several biological properties of these compounds were evaluated including their activity as direct 5-LO inhibitors in HEK293 cells, their capacity to inhibit LT biosynthesis in both human PMNL (important producers of the potent chemoattractant LTB_4_) and whole blood, their effects on the liberation of cellular arachidonic acid, and finally in their specificity for the 5-LO pathway versus the 12-LO pathway.

Molecular docking was undertaken with the help of AutoDock 4.0, Autogrid [33], and AutoDock Tools [34]. The “stable-5-LOX” (PDB ID: 3O8Y [34]) used for docking is mutated mostly on the noncatalytic domain with a small exception of a short 3 residues sequence mutated on the catalytic domain. While these mutations may affect the structure, test results showed that catalytic fidelity was conserved as “stable-5-LOX” activity was not affected [34]. While such docking results can be ambiguous in that they do not always reflect biological results, more molecular modeling with the tested molecules and others can nevertheless be helpful for the design of new analogs.

While most esters adopt a *cis* configuration, the docking pose of our best compound (**13**, Figure 3(a)) shows a *trans*-ester configuration. The small cavity, which is largely hydrophobic, the relatively large substituent (−CH_2_CH_2_CH_2_Ph), and the hydrogen bond completed with His367 (NH···O, 2.08 Å) can explain this *trans* conformation. As the pocket is relatively small and the substituent is relatively large, this limits the poses that the ligand can take. Similarly, both oxygens in the ester are in a small polar section of the pocket enabling the hydrogen bond and diminishing contact with the hydrophobic residues. For this hydrogen bond and positioning in the small polar pocket to happen, the ester needs to adopt a *trans* conformation to minimize steric blocking by Gln367 and Phe421. For the ligand to adopt a *cis* ester, a major shift would need to happen. Reported crystals of proteins include CAPE (**2**) (PDB ID: 4QRF), ferulic acid phenetyl ester (FAPE) (4PWF), ethyl caffeate (**4**) (4PWG), and other derivatives [40]. Of those three molecules, all crystalized in pairs, the pair of FAPE ligands have their ester in a *trans* configuration. One of the two ethyl caffeate is in a *trans* configuration while the second ethyl caffeate is in a *cis* configuration. As for CAPE (**2**), both deviate planarity with values of 38 degrees and 68 degrees. A recently described crystal structure with a pair of CAPE as ligands, (PDB ID: 5HNT), again, both deviate from planarity with angles of 53 degrees and 68 degrees.

In the first part of this study, the objective was to determine which compounds would potentially act as direct 5-LO inhibitors. HEK293 cells that expressed the necessary machinery to convert arachidonic acid into LTs [25, 41] were stimulated in the presence of exogenous arachidonic acid, thus bypassing a critical step in the bioavailability of the 5-LO substrate. In fact, exogenous substrate is required in this model since stimulated HEK293 cells do not use endogenous AA as a substrate for 5-LO [42, 43]. This strategy allowed for the identification of compounds that may affect the 5-LO pathway by directly inhibiting the enzyme. In addition to the reference molecules CAPE (**2**) and zileuton (**1**), most compounds significantly inhibited the biosynthesis of 5-LO products; however, the best compounds tended to be those from the series of aryl esters, consistent with the observation that the phenethyl ester moiety of CAPE is required for its inhibitory activity [31]. Accordingly, compound **9** that was designed as a mimic of CAPE (**2**) with a double bond and methyl group showed the best inhibitory activity amongst the compounds from the aliphatic series. Consistent with this model, the simplest aliphatic esters (**3**, **4**) were without inhibitory activity, while the only aryl compounds lacking activity were those devoid of a phenyl moiety suggesting that the interaction of the phenyl group with Phe421 stabilizes the compounds in the pocket.

Compound **13** was the only compound that showed significantly better inhibition than CAPE (**2**). The addition of a methylene group in compound **13** relative to CAPE (**2**) appears to be the determinant for the increased activity. This increase cannot be explained only by an enhancement of the lipophilic nature of the molecule since an extra CH_2_ does not greatly increase the lipophilicity. Better positioning of the molecule in the active site of 5-LO as shown with new interactions predicted by in silico modeling likely explains this increase in inhibitory activity. Conversely, the removal of methylene groups in **10** and **11** has the opposite effect resulting in molecules that are not as effective at inhibiting 5-LO as CAPE (**2**), suggesting that placement of the phenyl group in relation to the caffeoyl moiety is critical for optimal interactions and thus inhibitory activity.

While several compounds, including CAPE (**2**), appeared to perform better at inhibiting LT biosynthesis than the reference molecule zileuton in isolated cell assays, the inhibitory activity of zileuton (**1**) was similar to that of other compounds in the whole blood assay. This observation supports previous studies showing that zileuton (**1**) was a slightly better inhibitor of the biosynthesis of 5-LO products in whole blood than CAPE (**2**) [31]. At this time, the reasons for the better action of zileuton in a complex matrix are not known, but it could be due to tighter binding of the more lipophilic CAPE (**2**) analogues by plasma proteins, rendering the compounds less available for uptake into leukocytes, the target blood cells that are responsible for LT biosynthesis in this model [44]. This slightly decreased activity of CAPE and its analogues may also be attributed to the stability of the compounds in whole blood due to the presence of plasma esterases [45, 46]. Nevertheless, CAPE (**2**) and its analogues retained significant inhibitory activity in this complex matrix. Zileuton (**1**) on the hand, is less susceptible to plasma esterases as it lacks the ester moiety found in the test compounds. While CAPE's (**2**) bioavailability in humans has never been evaluated, pharmacokinetic studies have been performed in small rodents with an elimination half-life ranging from 21 min to 92 min in rats following IV administration [47, 48]. However, the pharmacokinetic profiles and bioavailability of polyphenols can vary considerably between humans and rodents, consequently establishing the bioavailability and pharmacokinetic profiles of our novel compounds in vivo is an essential step in the development of these potent 5-LO inhibitors.

Most of the compounds that exhibited 5-LO inhibition also inhibited the platelet 12-LO pathway. Compounds that display inhibitory properties for more than one inflammatory enzyme have attracted more attention in recent years [49, 50] due to their ability to simultaneously shut down multiple inflammatory pathways [9]. All of the compounds tested for the inhibition of the 12-LO pathway were inhibitory except compounds **16** and **19**, suggesting a degree of specificity for the 5-LO pathway. Therefore, the addition of an electron-donating group such as OCH_3_ in **16** does not seem to be favorable for the inhibition of 12-LO. The existence of unfavorable interactions with 12-LO may explain the loss of activity compared to CAPE (**2**) since both molecules have substantially the same lipophilicity. A significant increase in the lipophilicity, likely combined with new *π*-*π* interactions following the addition of a second phenyl as in **19** also appears to hinder the ability of this compound to inhibit the 12-LO.

Since both CAPE (**2**) and zileuton (**1**) are known to interfere with the release of arachidonic acid from cell membranes, likely via a mechanism inhibiting the group IVA cytosolic phospholipase A_2_ (cPLA_2_*α*) [31, 38], we investigated whether our compounds displayed similar biological properties. While **9**, **13**, **19**, and CAPE (**2**) all inhibited the release of cellular arachidonic acid, compounds **14**, **15**, and **16** bearing an extra methyl, fluorine, or a methoxy, respectively, in the para position did not interfere with AA liberation. Although the inhibition of AA release contributes to the inhibition of LT biosynthesis, all compounds nevertheless show inhibition of 5-LO activity as demonstrated in the experiments in HEK293 cells where exogenous substrate was provided to the cells. Finally, these series of compounds all exhibit antioxidant properties [24], which contributes to their inhibitory characteristics on enzymes such as lipoxygenases; however, their ability to inhibit at the concentrations used in the present study are nevertheless dependent on the presence and the nature of the ester linkage [31].

While leukotrienes are necessary for host defense against pathogens, they also participate in chronic inflammatory diseases such as asthma, arthritis, and atherosclerosis. For years, researchers have targeted 5-LO as a means of regulating the biosynthesis of leukotrienes. To this day, zileuton remains the only clinically used and approved 5-LO inhibitor but its usage has been linked to severe side effects in some patients. In this study, we investigated structural analogues of caffeic acid phenethyl ester, a 5-LO inhibitor recently described by our team, in an attempt to enhance the inhibitory activity against 5-LO and determine structure-activity relationships. These compounds were investigated for their ability to attenuate the biosynthesis of leukotrienes either as direct 5-LO inhibitors or preventing its substrate's release from cellular membranes. Interestingly, a phenpropyl ester (**13)** and a diphenylethyl ester **(19)** were identified that exhibited potent inhibitory activity when compared to the reference molecules caffeic acid phenethyl ester and zileuton. In the search for better 5-LO inhibitors, we provide a better understanding of the fundamental mechanism by which these molecules exhibit anti-inflammatory properties, an important step in the development of novel therapeutic drugs.

## Supplementary Material

Figure S1: Annexin V labelling was performed on PMNL following 20 min incubation at 37°C in the presence of diluent (control, DMSO 0.5%) or compound 13 that showed the best inhibition of 5-LO product biosynthesis. A positive control with 10% EtOH is shown to validate the assay.

## Figures and Tables

**Table 1:** Calculated IC_50_ values of selected compounds for the inhibition of 5-LO product biosynthesis in human PMNL.

Compound	IC_50_ (*μ*M) (95% CI)
CAPE (**2**)	0.79 (0.65–0.97)
**9**	0.87 (0.70–1.11)
**13**	0.49 (0.41–0.58)
**14**	1.01 (0.69–1.50)
**15**	1.04 (0.30–3.65)
**16**	1.29 (0.83–2.00)
**19**	0.53 (0.41–0.68)
Zileuton (**1**)	1.9 (1.48–2.42)^∗^

CI = 95% confidence intervals; ND = IC_50_ values could not be determine at tested concentrations used for the analysis; ^∗^IC_50_ values taken from [31].

**Table 2:** Ligand binding energy, interactions, and hydrogen bond lengths.

Molecule	Binding energy (kcal/mol)	Hydrogen bonds	Hydrogen bonds length (Å)	*π*-*π* Interactions
CAPE (**2**)	−8.19	His367, Asn407 × 2	2.14, 2.21, 2.26	Phe421
Zileuton (**1**)	−7.18	Gln363, His367	2.09, 2.11	Phe177
(**3**)	−6.78	Ala424	1.81	None
(**4**)	−6.15	Tyr181, Gln363 × 2, Asn425	2.25, 1.98, 1.94, 2.21	None
(**5**)	−6.63	Tyr181, Gln363, Asn425	2.02, 1.96,2.01	None
(**6**)	−6.84	Tyr181, Gln363, Asn425	2.15, 1.94, 1.95	None
(**7**)	−7.12	Tyr181, Gln363, Asn425	2.48, 1.99, 1.95	None
(**8**)	−7.22	Tyr181, Gln363, Asn425	1.98, 2.09, 2.00	None
(**9**)	−7.21	Tyr181, Gln363, Asn425	2.08, 2.02, 1.93	None
(**10**)	−8.23	Tyr181, Gln363, Asn425	1.99, 2.04, 1.91	Tyr181
(**11**)	−7.96	Tyr181	2.04	Tyr181
(**12**)	−8.69	His367, Asn407 × 2	2.01, 2.22, 2.30	None
(**13**)	−8.77	His367, Asn407 × 2	2.08, 2.11, 2.27	None
(**14**)	−8.04	His367, Ile406	2.10, 2.26	Phe421
(**15**)	−8.77	His367, Asn407 × 2	2.11, 2.14, 2.45	Tyr181, Phe421
(**16**)	−8.69	His367, Asn407 × 2	2.14, 2.21, 2.32	Phe421
(**17**)	−10.91	His367, Asn407 × 2, Ala424	2.50, 2.07, 2.32, 1.99	Phe177, Phe421
(**18**)	−10.62	His367, Asn407 × 2	2.34, 2.07, 2.22	Phe177
(**19**)	−9.14	Leu420	2.61	Tyr181, Phe421
