# Supplementary material for: New Hydroxycinnamic Acid Esters as Novel 5-Lipoxygenase Inhibitors That Affect Leukotriene Biosynthesis
Source: Mediators Inflamm. 2017 Jun 7;2017:6904634. doi: 10.1155/2017/6904634 (PMC5478869; doi:10.1155/2017/6904634)
Supplement: Supplementary file 2 [file 6904634.f2.pdf]

New hydroxycinnamic acid esters as novel 5-lipoxygenase inhibitors that affect leukotriene biosynthesis

Luc H. Boudreau, Grégoire Lassalle-Claux, Marc Cormier, Sébastien Blanchard, Marco S. Doucet, Marc E. Surette and Mohamed Touaibia.
Department of Chemistry and Biochemistry, Université de Moncton, Moncton, New Brunswick, Canada E1A 3E9.
Correspondence should be addressed to Mohamed Touaibia: mohamed.touaibia@umoncton.ca

Supplementary Figure 1

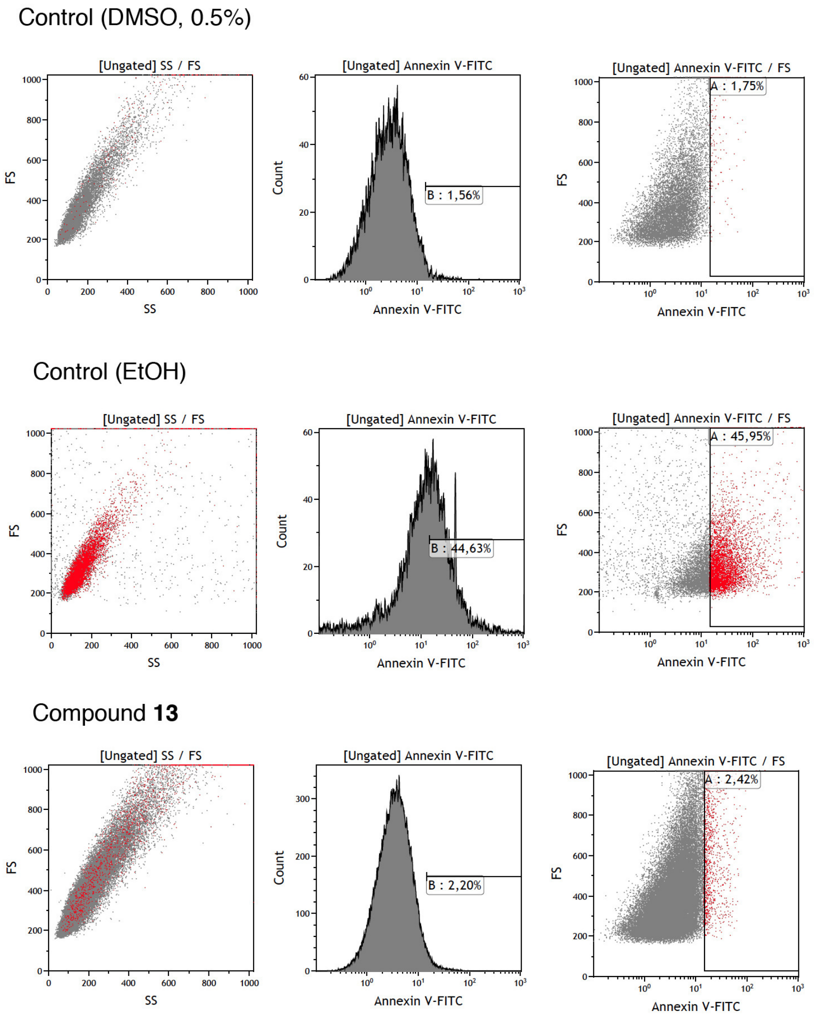

Figure S1: Annexin V labelling was performed on PMNL following 20 min incubation at 37°C in the presence of diluent (control, DMSO 0.5%) or compound **13** that showed the best inhibition of 5-LO product biosynthesis. A positive control with 10% EtOH is shown to validate the assay.
